# Cumulative Risks of Foster Care Placement by Age 18 for U.S. Children, 2000–2011

**DOI:** 10.1371/journal.pone.0092785

**Published:** 2014-03-26

**Authors:** Christopher Wildeman, Natalia Emanuel

**Affiliations:** 1 Department of Sociology, Yale University, New Haven, Connecticut, United States of America; 2 Department of Economics, Yale University, New Haven, Connecticut, United States of America; Wayne State University, United States of America

## Abstract

Foster care placement is among the most tragic events a child can experience because it more often than not implies that a child has experienced or is at very high risk of experiencing abuse or neglect serious enough to warrant state intervention. Yet it is unclear how many children will experience foster care placement at some point between birth and age 18. Using synthetic cohort life tables and data from the Adoption and Foster Care Analysis and Reporting System (AFCARS), we estimated how many U.S. children were placed in foster care between birth and age 18, finding support for three conclusions. First, up to 5.91% of all U.S. children were ever placed in foster care between their birth and age 18. Second, Native American (up to 15.44%) and Black (up to 11.53%) children were at far higher risk of placement. Foster care is thus quite common in the U.S., especially for historically disadvantaged racial/ethnic groups. Third, differences in foster care placement were minimal between the sexes, indicating that the high risks of foster care placement are shared almost equally by boys and girls.

## Introduction

Of all childhood experiences, foster care placement is among the most tragic. In most instances, children are placed in foster care because the state rules that the child has experienced or is at high risk of experiencing maltreatment (abuse or neglect). Though clearly helpful to some children, foster care placement frequently introduces additional instability to their already-chaotic lives, potentially further harming them. This combination of maltreatment and instability means that children who have experienced foster care suffer not only from elevated rates of mortality in childhood [1], but also from a host of other problems ranging from asthma to behavioral problems to suicidal ideation [2]–[9]. Children in foster care are five times more likely to be diagnosed with depression, four times more likely to be diagnosed with ADHD, and ten times more likely to be diagnosed with bipolar disorder than other children, for instance [9].

Annual foster care entry rates and point-in-time estimates of the number of children in foster care suggest that foster care placement is rare. In 2011, the most recent year for which data are available, only 0.34% of all American children entered foster care within the year, and only 0.54% of children were in foster care on any given day [10]. These estimates imply that despite foster care placement's implications for individual children, its societal importance may be minimal because it affects few children. However, these data may create an inaccurate portrayal of how common foster care placement is because annual and daily estimates of children in foster care do not convey how many children ever experience placement during their entire childhood.

Is foster care placement an event so uncommon it requires minimal consideration? Or, is it an event common enough that it merits serious attention from researchers and policymakers? We provide insight into these questions by using synthetic cohort life tables to estimate the cumulative probability of foster care placement for children from birth to age 18 with data from the Adoption and Foster Care Analysis and Reporting System (AFCARS) in the years 2000–2011. We also stratify the risk of being placed in care by age 18 by race/ethnicity, sex, and year.

## Materials and Methods

### The Adoption and Foster Care Analysis and Reporting System (AFCARS)

We used data from the Adoption and Foster Care Analysis and Reporting System (AFCARS), which includes all children in foster care from 2000–2011, for the numerator [11]–[22], in concert with race-, age-, sex-, and year-specific estimates of the U.S. population for the denominator [23]. The number of children at risk of experiencing their first foster care placement by age and year is recorded in Table 1. The number of children experiencing their first foster care placement by age and year is recorded in Table 2.

**Table 1:** Number of Children at Risk of First Foster Care Placement by Age (0–17) and Year (2000–2011).

	Year											
Age	2000	2001	2002	2003	2004	2005	2006	2007	2008	2009	2010	2011
0	3817337	3977005	3988728	4011594	4066140	4095537	4138834	4249613	4270595	4261405	4005929	3821941
1	3764118	3816152	3962810	3971289	3989636	4053455	4068708	4112274	4225764	4257656	3980460	3759528
2	3727845	3757609	3822725	3967012	3970974	3996182	4043332	4065972	4116692	4240388	4069027	3777846
3	3740710	3750272	3763253	3826872	3965441	3972760	3987987	4042831	4071688	4133576	4044219	3860081
4	3807201	3770357	3755410	3767854	3826276	3961967	3968119	3990361	4050874	4091165	3994862	3854939
5	3866721	3834668	3776029	3760101	3766662	3831820	3967335	3973879	4000212	4075110	3977485	3816203
6	3871413	3880311	3839153	3780808	3759747	3776277	3830330	3971655	3985377	4022503	3960894	3800271
7	3961367	3917585	3884610	3844236	3779481	3770867	3771644	3833995	3982894	4005970	3923837	3789753
8	4021847	3987541	3921811	3889423	3842584	3778579	3760959	3775298	3847287	4003076	3918452	3757346
9	4076559	4048141	3991936	3926851	3888051	3837712	3779985	3770005	3790699	3868190	3947180	3762875
10	4080531	4107297	4052590	3997126	3925038	3881198	3843088	3791529	3786009	3817307	3970711	3839999
11	3948861	4113302	4112290	4058276	3995212	3916391	3888012	3856167	3808186	3812215	3911544	3860412
12	3884542	3969450	4118535	4117784	4055709	3985423	3924600	3901982	3873449	3836472	3908685	3804025
13	3830493	3898127	3974856	4123433	4114125	4044305	3993900	3938575	3918841	3901364	3926078	3798243
14	3840951	3858315	3904491	3979710	4118947	4100735	4052337	4007647	3955705	3947867	3952525	3802568
15	3833648	3860880	3865312	3910429	3976812	4104834	4110818	4067093	4025580	3986792	4013589	3833816
16	3762243	3837833	3871121	3874454	3909072	3972962	4119745	4127368	4086973	4058751	4083641	3901616
17	3809558	3786011	3857722	3887789	3878584	3919094	3988588	4139415	4152137	4122481	4136260	3964276

**Table 2:** Number of Children Experiencing First Foster Care Placement by Age (0–17) and Year (2000–2011).

	Year											
Age	2000	2001	2002	2003	2004	2005	2006	2007	2008	2009	2010	2011
0	34587	36136	38098	38902	41343	45162	46064	46353	42838	39145	38912	37012
1	13940	15038	15687	15719	16579	18205	18183	18049	17282	16820	16858	16432
2	11997	13466	13471	13892	14397	15449	15413	15016	14227	14082	14609	14254
3	10614	11797	12021	12130	13064	13628	13387	12954	12168	11428	12395	12765
4	9716	10543	10716	10924	11428	12202	12033	11502	10596	10127	10535	11246
5	9594	9931	10196	9870	10712	11330	11385	10697	9693	8991	9462	9696
6	9752	9990	9516	9467	9861	10613	10440	10071	9078	8297	8583	8753
7	9420	9749	9244	9025	9169	9449	9703	9078	8410	7685	7807	7763
8	9232	9732	9120	8529	8618	8708	8791	8397	7566	7016	6957	6971
9	9157	9487	9038	8535	8212	8146	8115	7642	7224	6438	6526	6647
10	8736	9415	8974	8368	8436	8020	7711	7167	6539	6143	6227	6122
11	8546	9335	9118	8920	8602	8477	7734	7155	6492	6051	6110	5939
12	9583	10306	9851	9942	10040	9534	8695	7926	7150	6556	6558	6458
13	11576	12157	11985	11928	11944	11772	10734	9739	9011	7823	7726	7690
14	14312	14203	14097	13794	14063	14136	13277	12513	11288	10036	9338	9132
15	15104	15967	15385	14928	15573	15657	15626	14764	13587	12374	11237	10641
16	12717	13596	13362	12954	13454	14160	14030	13638	13167	12188	11127	10578
17	7166	7498	7334	7331	7595	7835	8247	8063	8070	7818	7258	6830

The AFCARS data are publicly available through the National Data Archive on Child Abuse and Neglect, which is housed at Cornell University, and have been de-identified prior to being made available to researchers in the publicly available version of the data, which are the data we used for all analyses. These data can be accessed after signing a terms of use agreement form here: http://www.ndacan.cornell.edu/datasets/datasets-list-afcars.cfm. Because the Adoption and Foster Care Analysis and Reporting System (AFCARS) data are publicly available and de-identified, the Yale University Institutional Review Board deemed this research exempt.

The AFCARS data contain “case-level information on all children in foster care for whom State and Tribal title IV-E agencies have responsibility for placement, care or supervision and on children who are adopted under the auspices of the State and Tribal title IV-E agency. Title IV-E agencies are required to submit AFCARS data semi-annually to the Children's Bureau. The AFCARS report periods are October 1 through March 31 and April 1 through September 30” [24]. We used the combined reporting files, so what we refer to as 2010 spans October 1, 2009 to September 30, 2010. Because reporting to the AFCARS is mandatory, all states contributed data for all 12 years.

The AFCARS data include information pertinent to the child welfare system, such as when the child was most recently placed in foster care and whether that was the first placement or a higher order placement, as well as basic demographic information such as age, sex, and race. Additionally, the dataset includes information on how long the child has been in care—a number that ranges from a few days to many years—and what type of care arrangement they are in, specifying, for instance, whether the child is living in a pre-adoptive home, kinship foster care, non-kin foster care, a group home, an institution including a juvenile detention center (although children in a juvenile detention center would only be considered to be in foster care if removed from their homes for one of the reasons listed below), or independently.

Roughly two-thirds of the children in the data enter foster care because they experienced maltreatment, with the other one-third of children entering foster care for a variety of reasons tied to their parents. Such reasons include parental drug or alcohol abuse, parental death, parental inability to cope (encompassing many things), parental abandonment, and inadequate housing. Some children were admitted for reasons tied to the child, including having disabilities better provided for in foster care or exhibiting serious behavioral problems.

### Measures

We rely on four measures: (1) age, (2) sex, (3) race/ethnicity, and (4) first admission to foster care. Age at first admission to foster care is based on the difference between the admission date and his/her birth date. Sex is based on caseworker reports of sex. There is little missing data on age or sex; in 2005, 915 of about 800,000 cases, or 0.1%, were missing data on sex or age. We treated these cases as missing completely at random. Because of the small amount of missing data (∼0.1%), the choice of method for dealing with it minimally affects the results.

Race/ethnicity is based on caseworker reports, with Asian, Black, Hispanic, Native American, Pacific Islander, and White as the options. Some children were reported as having more than once race, but since we wanted to provide estimates for specific racial/ethnic groups, we were forced to assign them one racial/ethnic identity. In all cases in which the caseworker reported the child was Native American, we considered the child to be Native American (regardless of additional identities). For those children who were not Native American but were considered to be multiracial, we considered (1) children reported to have Hispanic ethnicity as Hispanic, (2) children reported to be Black (but not Hispanic) as Black, (3) children reported to be Asian or Pacific Islander (but not Hispanic or Black) as Asian, and (4) all remaining children as White. To account for the approximately 2% of cases who had missing racial/ethnic identity information we distributed these cases in the following way. Children whose Hispanic ethnicity was marked “unable to determine” and who had missing racial data were distributed amongst Hispanic cases; children whose race was marked “unable to determine” and whose ethnicity was unmarked were distributed amongst non-White cases; and children for whom both racial and ethnic data was missing were distributed amongst all cases equally. Because of the small amount of missing data (∼2%), the choice of method for dealing with it minimally affects the results.

Our measure of first time admissions to foster care in the last year is based on whether the admission was the first placement to foster care and occurred in the reporting year and is based on a constructed variable in the dataset that allowed us to differentiate first and subsequent admissions by matching children based on unique (de-identified) IDs. This measure is crucial for our analysis because if we also included subsequent admissions to foster care, we would count children as experiencing the event more than once, thereby incorrectly inflating the estimates.

### Analytic Strategy

To construct the estimates of the cumulative risk of foster care placement between birth and age 18, we use synthetic cohort (or period) life tables. Synthetic cohort life tables, which were originally designed to study mortality, have long been used to study what proportion of a hypothetical cohort of 100,000 individuals would survive to any given age if they were exposed to any year's age-specific mortality rates at each age. (The Census Bureau still uses this method to estimate life expectancy at birth in the United States.) In this adaptation, the synthetic cohort life table provides an estimate of the proportion of a hypothetical cohort of children exposed to the age-specific first time foster care admission rates at each age who could expect to experience foster care placement at some point between birth and age 18 [25].

The key benefit of synthetic cohort life tables for our analysis is that they allow us to estimate the cumulative risk of foster care placement to age 18 using only 1 year of data, a calculation that would be impossible using birth cohort life tables, which require 18 years of data to provide that estimate because they follow a birth cohort through time.

Synthetic cohort life tables also have significant limitations. Most importantly, the estimates they produce may be unreliable when the age-specific rates are changing quickly. This limitation is especially problematic if the number of synthetic cohorts estimated is small. Although this can be a key limitation of this method, it is important to note two things before reviewing the results. First, with one exception (Native Americans), the age-specific risks of foster care placement are changing gradually, not rapidly, in our data, meaning that the bias in our estimates should be minimal. Second, because we have 12 years of data, it would be easy to identify years in which the estimates were unreliable. Indeed, parallel analyses comparing synthetic and birth cohort life tables estimating the cumulative risk of foster care placement in California showed that even with only two synthetic cohorts (based on 2000 age-specific first-time entry rates and 2006 age-specific first-time entry rates), estimates using synthetic cohort and birth cohort life tables were very similar [26].

Because children who have already been placed in the foster care system are still included in the total population counts provided by CDC Wonder (even though they are no longer at risk of first placement), we adjust the denominator down accordingly. (Table 1 presents the adjusted denominators by age.) For example, we multiply the number of children five years of age in the population in 2005 by the probability of having never been placed in foster care by age five based on 2005 rates for children five years of age. This adjustment, although important for the precision of the results, does not greatly alter the findings.

Properly counting children who enter the population of children at risk of first foster care placement (through immigration) and who leave the population of children at risk of first foster care placement (through emigration and death) is also essential. Because the number of children at risk of first foster care placement is updated annually (based on the number of children in the population according to CDC Wonder), children entering and leaving the population at risk only minimally affect our results.

Because synthetic cohort life tables rely on only one year of data, we provided annual estimates of the cumulative risk of foster care placement for each year from 2000 to 2011. Though our data include the entire population of interest, we present confidence intervals because even in the most complete dataset, there is always some disparity from the population. Our confidence intervals are based on Greenwood's formula for the asymptotic standard error [27]. We used Stata/SE 12 for all analyses [28].

## Results

The cumulative risk of ever being placed in foster care between birth and age 18 for all American children was 5.91% in 2005 (Table 3). By age six, the cumulative risk of foster care placement was 3.11% (Table 3). This risk increased to 4.42% by age 12 and 5.91% by age 18 (Table 3).

**Table 3:** Cumulative Risks of Foster Care Placement from Birth to Age 18 for All U.S. Children and White, Black, Hispanic, Asian, and Native American Children, 2005.

Age	All U.S. Children (95% CI)		White (95% CI)		Black (95% CI)		Hispanic (95% CI)		Asian (95% CI)		Native American (95% CI)	
0	**0.011**	(0.011, 0.011)	**0.009**	(0.009, 0.009)	**0.023**	(0.023, 0.024)	**0.009**	(0.009, 0.009)	**0.004**	(0.003, 0.004)	**0.030**	(0.028, 0.032)
1	**0.015**	(0.015, 0.016)	**0.012**	(0.012, 0.013)	**0.032**	(0.031, 0.032)	**0.013**	(0.012, 0.013)	**0.005**	(0.005, 0.005)	**0.043**	(0.041, 0.045)
2	**0.019**	(0.019, 0.019)	**0.016**	(0.015, 0.016)	**0.038**	(0.038, 0.039)	**0.016**	(0.016, 0.016)	**0.006**	(0.006, 0.007)	**0.055**	(0.053, 0.058)
3	**0.023**	(0.022, 0.023)	**0.019**	(0.018, 0.019)	**0.044**	(0.044, 0.045)	**0.019**	(0.019, 0.019)	**0.007**	(0.006, 0.007)	**0.066**	(0.063, 0.069)
4	**0.026**	(0.025, 0.026)	**0.021**	(0.021, 0.021)	**0.050**	(0.049, 0.050)	**0.022**	(0.021, 0.022)	**0.008**	(0.007, 0.008)	**0.076**	(0.073, 0.079)
5	**0.028**	(0.028, 0.029)	**0.023**	(0.023, 0.024)	**0.055**	(0.054, 0.056)	**0.024**	(0.024, 0.025)	**0.009**	(0.008, 0.009)	**0.085**	(0.082, 0.088)
6	**0.031**	(0.031, 0.031)	**0.026**	(0.025, 0.026)	**0.060**	(0.059, 0.060)	**0.027**	(0.027, 0.028)	**0.010**	(0.009, 0.010)	**0.092**	(0.089, 0.095)
7	**0.034**	(0.033, 0.034)	**0.028**	(0.027, 0.028)	**0.064**	(0.063, 0.065)	**0.030**	(0.029, 0.030)	**0.011**	(0.010, 0.011)	**0.099**	(0.096, 0.102)
8	**0.036**	(0.036, 0.036)	**0.029**	(0.029, 0.030)	**0.068**	(0.067, 0.069)	**0.032**	(0.031, 0.032)	**0.011**	(0.011, 0.012)	**0.105**	(0.102, 0.109)
9	**0.038**	(0.038, 0.038)	**0.031**	(0.031, 0.031)	**0.072**	(0.071, 0.073)	**0.034**	(0.033, 0.034)	**0.012**	(0.012, 0.013)	**0.111**	(0.107, 0.114)
10	**0.040**	(0.040, 0.040)	**0.033**	(0.032, 0.033)	**0.075**	(0.075, 0.076)	**0.036**	(0.035, 0.036)	**0.013**	(0.012, 0.014)	**0.116**	(0.113, 0.120)
11	**0.042**	(0.042, 0.042)	**0.034**	(0.034, 0.035)	**0.079**	(0.078, 0.080)	**0.037**	(0.037, 0.038)	**0.014**	(0.013, 0.015)	**0.121**	(0.118, 0.125)
12	**0.044**	(0.044, 0.044)	**0.036**	(0.036, 0.036)	**0.083**	(0.083, 0.084)	**0.039**	(0.039, 0.040)	**0.015**	(0.014, 0.016)	**0.128**	(0.124, 0.131)
13	**0.047**	(0.047, 0.047)	**0.038**	(0.038, 0.039)	**0.089**	(0.088, 0.090)	**0.042**	(0.041, 0.042)	**0.016**	(0.015, 0.017)	**0.134**	(0.130, 0.137)
14	**0.050**	(0.050, 0.051)	**0.041**	(0.041, 0.041)	**0.094**	(0.094, 0.095)	**0.045**	(0.044, 0.046)	**0.017**	(0.017, 0.018)	**0.140**	(0.136, 0.144)
15	**0.054**	(0.054, 0.054)	**0.044**	(0.044, 0.044)	**0.101**	(0.100, 0.102)	**0.048**	(0.048, 0.049)	**0.019**	(0.018, 0.020)	**0.147**	(0.143, 0.150)
16	**0.057**	(0.057, 0.058)	**0.047**	(0.047, 0.047)	**0.107**	(0.106, 0.108)	**0.052**	(0.051, 0.052)	**0.020**	(0.020, 0.021)	**0.152**	(0.148, 0.156)
17	**0.059**	(0.059, 0.059)	**0.049**	(0.048, 0.049)	**0.110**	(0.109, 0.111)	**0.054**	(0.053, 0.054)	**0.021**	(0.021, 0.022)	**0.154**	(0.151, 0.158)

Cumulative risks of foster care placement differed dramatically by race/ethnicity. White and Hispanic children had cumulative risks of foster care placement relatively close to those for the population, at 4.86% and 5.35%, respectively (Table 3). In contrast, Asian children had the lowest risk at 2.14%, while Black children and Native American children had the highest cumulative risks of placement, at 10.99% and 15.44%, respectively (Table 3). Compared to White children, Hispanic children were at 1.10 relative risk of foster care placement, Asian children were at 0.44 relative risk of foster care placement, Black children were at 2.26 relative risk of foster care placement, and Native American children were at 3.18 relative risk of foster care placement (*p*<.001 for all comparisons to the White population; see Table 3 for 95% Confidence Intervals).

**Figure 1:**
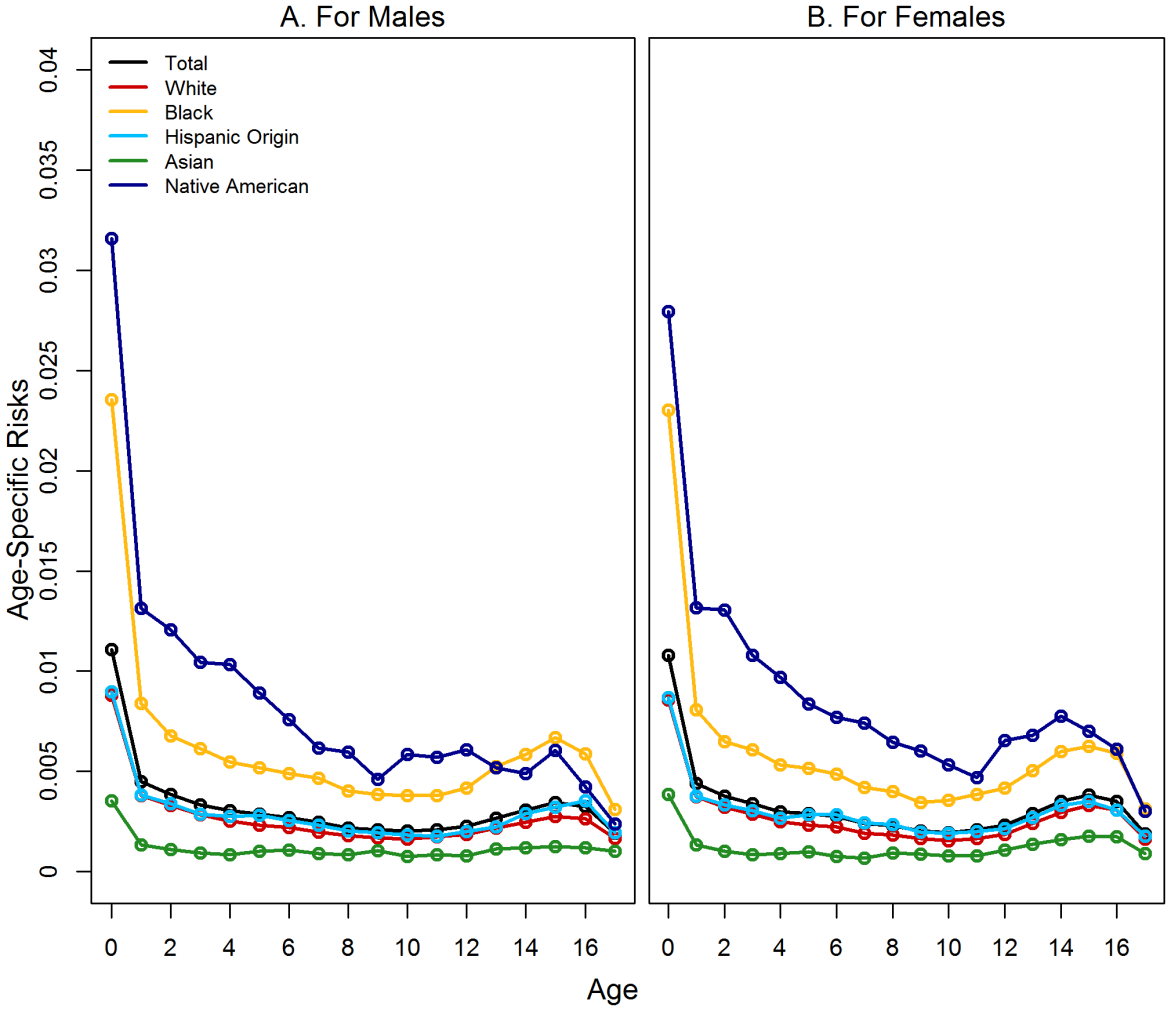
Age-Specific Risks of First-Time Foster Care Placement.

Children had the highest risk of first foster care placement during infancy, with 1.09% of all U.S. children first entering care before their first birthday (Figure 1). The risk of first placement then trailed off until age 13, at which point it increased throughout adolescence. The age-patterning of first placement was similar for all groups (Figure 1).

**Figure 2:**
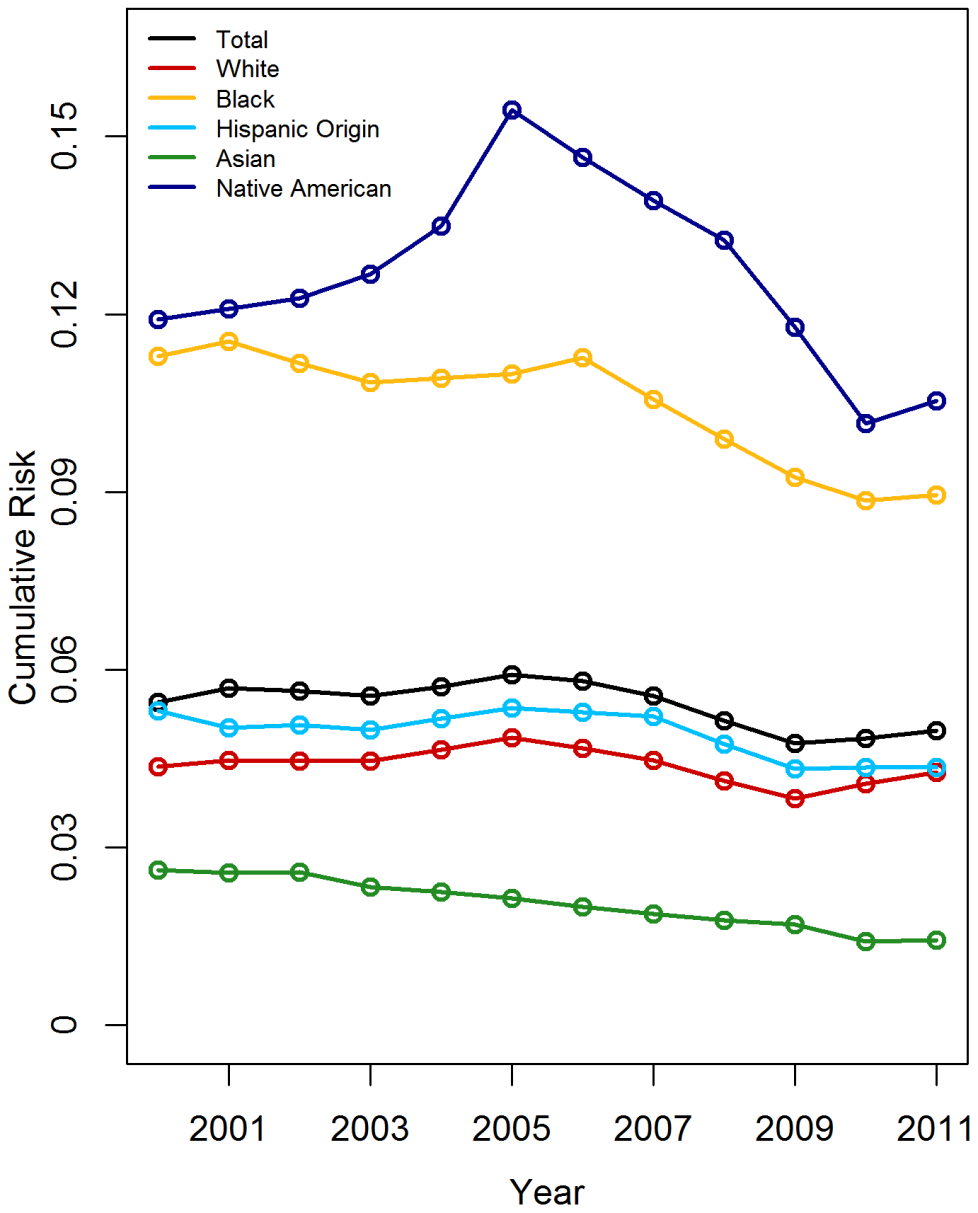
Cumulative Risk of Foster Care Placement by Age 18.

Between 2000 and 2011, cumulative risks of foster care placement declined slowly, but substantial racial/ethnic disparities persisted (Figure 2). The percentage of all U.S. children estimated to ever be in foster care between birth and age 18 ranged from 4.76% in 2009 to 5.91% in 2005 (Figure 2). Shifts were largest for Native American children, whose risk ranged from 10.54% in 2011 to 15.44% in 2005, and Black children, whose risk ranged from 8.84% in 2010 to 11.53% in 2001.

**Figure 3:**
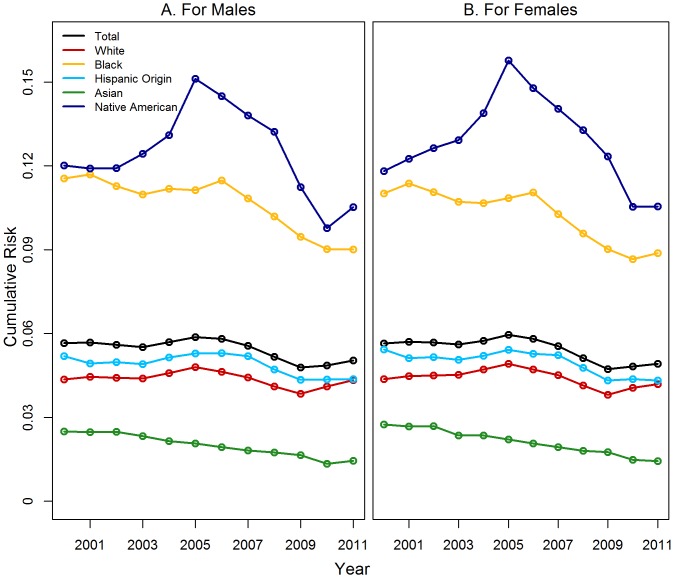
Cumulative Risk of Foster Care Placement by Demographic Group.

Females were more likely to ever be placed in foster care than were males for all years, but these differences were often small (Figure 3). Thus, racial/ethnic stratification in the cumulative risk of placement was more substantial than was sex stratification.

## Discussion

The results from our analyses demonstrate that foster care placement is far more common than often thought. Up to 5.91% of U.S. children (1 in 17) will experience foster care placement at some point between birth and age 18. The risk, however, is not evenly distributed. A shocking 15.44% (1 in 7) of Native American children and 11.53% (1 in 9) of Black children will enter foster care at some point before they turn 18. This risk of being in foster care is shared almost equally by boys and girls, further suggesting the global nature of the problem.

Although this is not the first study to use demographic methods to estimate the cumulative risk of foster care placement [26], it nonetheless extends research in this area in three key ways. First, it considers the entire country instead of just one state (California), giving broad insight into how common foster care placement is across the entire United States. Second, it provides cumulative risk estimates throughout the entirety of childhood (to age 18) rather than to age 7. Finally, it provides estimates for the entire period during which U.S. foster care caseloads have declined so sharply (2000–2011) rather than just during the early part of this period (2000–2006). Thus, the current study greatly advances research beyond the important early applications of life tables to study the cumulative risk of foster care placement to age seven in California [26].

These findings document a pressing need for further research and policy measures on the topic. The prevalence of foster care in the lives of American children, for instance, suggests that further investigation into the *consequences* of placement in foster care—as distinct from the circumstances that lead to foster care placement—and how foster care can be more beneficial to children is necessary. Moreover, since foster care placement is indicative of poor life circumstances—whether because of the conditions that caused it or because of the instability resulting from removal from one's birth family—it is concerning that Black and Native American children have far greater risks of experiencing such circumstances. Such findings call for additional research on the interaction between social inequality and foster care placement. Finally, since the risk of placement is highest in the first year of life, additional support to pregnant women and first time mothers may be good policy to reduce foster placements.

In light of these high cumulative foster care placement risks and associated outcomes, researchers and policymakers must give far greater attention to this vulnerable group of children.
